# Experimental data for synthesis of bi-metalized chitosan particle for attenuating of an azo dye from wastewater

**DOI:** 10.1016/j.dib.2016.01.066

**Published:** 2016-02-10

**Authors:** Abdollah Hajivandi, Sima Farjadfard, Bahman Ramavandi, Samad Akbarzadeh

**Affiliations:** a Department of Biostatistics, Faculty of Health, Bushehr University of Medical Sciences, Bushehr, Iran; b Department of Environmental Engineering, Graduate School of the Environment and Energy, Science and Research Branch, Islamic Azad University, Tehran, Iran; c Environmental Health Engineering Department, Faculty of Health, Bushehr University of Medical Sciences, Bushehr, Iran; d Systems Environmental Health, Oil, Gas and Energy Research Center, Bushehr University of Medical Sciences, Bushehr, Iran; e Department of Biochemistry, The Persian Gulf Biotechnology Research Center, Bushehr University of Medical Sciences, Bushehr 7514763448, Iran

**Keywords:** Decolorization, Bi-metalized chitosan particle, Adsorption, Orange II dye, Textile wastewater

## Abstract

In this data article, we introduce data acquired from new adsorbent, bi-metalized chitosan particle that is successfully synthesized and applied to remove the orange II dye, an azo dye, from textile wastewater. The adsorbent was meso- and macro-porous material with BET surface area of 12.69 m^2^/g and pH_zpc_ 6.6. The simulated textile-wastewater can be significantly treated using a relatively low quantity of the adsorbent. Overall, the use of bi-metalized chitosan particle can be considered a promising method for eliminating the azo dye from wastewater effectively. Accordingly, these data will be useful for decolorizing of azo dyes from textile wastewater.

**Specifications Table**

**Table:** 

Subject area	Chemical Engineering
More specific subject area	Environmental remediation
Type of data	Table, image, and figure
How data was acquired	– orange II dye concentration removal efficiency was determined based on orange II dye residue content in the centrifuged solution – pH meter (METLER TOLEDO FE20), transmission electron microscope (TEM) (FEI Tecnai G2 20S TWIN, USA).
Data format	Analyzed
Experimental factors	Bi-metalization of chitosan particle for azo dye removal
Experimental features	– Chitosan: The chitosan was prepared from shrimp (*Philocheras lowisi*) shell wastes. – Bi-metalized chitosan particle: Bi-metalization of chitosan by Cu/Mg particles was provided by using a modified water-based approach. – The bi-metalized chitosan particle was used for attenuating orange II dye from a textile wastewater. – The data related to characteristics of the adsorbent was acquired.
Data source location	Bushehr University of Medical Sciences, Bushehr, Iran, GPS: 28.9667°N, 50.8333°E
Data accessibility	Data are available with the article

**Value of the data**

• A simple method used for producing of bi-metalized chitosan particles.
• The article describes the preparation and characterization of bi-metalization of chitosan particles.
• The method of bi-metalization of chitosan could be useful approach for treating wastewater containing azo dyes.

## 1 Data

Data presented here describes the bi-metallic chitosan particles which synthesized by mixing a solution of copper ion with Mg°–chitosan particles. The bi-metalized chitosan particles were applied for attenuating of an azo dye (orange II) from a textile wastewater. The dye was adsorbed to the bi-metalized chitosan particles as an adsorbent by protonation of the azo dye.

## 2 Experimental design, materials and methods

### 2.1 Materials

All the chemicals used in the tests were of analytical grade and purchased from Merck (Darmstadt, Germany). The orange II dye was purchased from Fluka (Buchs, Switzerland). The dye content was 95%, and the solutions were prepared accordingly. The orange II properties are listed in Table 1 [1], [2].

### 2.2 Extraction of chitosan from shrimp waste

The shell wastes of a given shrimp (*Philocheras lowisi*) which directly collected from the Persian Gulf, was used for extracting of the chitosan. First, the shells were rinsed and then submerged in 10 wt% NaOH for 120 min with agitation to remove proteins (20% w/v); in 1.8 mol/L of HCl for 12 h to remove calcium minerals (25% w/v), and in 0.38 wt% NaClO for 30 min with agitation to remove pigments (25% w/v). The product (chitin) was deacetylated in 50 wt% NaOH for 60 min at 110 °C (15% w/v). The deacetylation was done according to method stated by No and Meyers [3] and Novikov [4]. The mixture was then rinsed with distilled water to remove the residual sodium hydroxide. The product (chitosan) was dried at 50 °C for 8 h and finally sieved for modification by bimetal particles.

### 2.3 Bi-metallization of chitosan

The bi-metallization of chitosan by Cu/Mg particles was provided by using a modified water-based approach. The preparation was performed in a 250-mL flask attached to a vacuum line. Particularly, a given amount of chitosan particles was soaked in distilled water and was then purged with purified N_2_ for 45 min to remove dissolved oxygen. In a typical preparation, first, a solution containing 0.214 M MgCl_2_·6H_2_O was prepared before use and then added to the chitosan solution to give a desired concentration of Mg^2+^ and chitosan. The mixture was purged with N_2_ in an ultrasonic bath for around 1 h to ensure the complete formation of the Mg^2+^–chitosan complex. Second, the Mg^2+^ ions were reduced to Mg° by adding a given amount of sodium borohydride (BH_4_^−^/Mg^2+^=2) drop wise to the above Mg^2+^–chitosan solution under inert conditions through continuous vacuuming.

Then, the bi-metallized chitosan particles were synthesized by mixing a copper ion solution with Mg°–chitosan particles. The copper ion solution was prepared by dissolving CuCl_2_ in distilled water. The bi-metallized chitosan was provided using copper bulk loadings of 1 wt% by diluting the desirable amount of the copper ion solution to 100 mL with distilled water and then adding this solution to 10 g of fresh Mg°–chitosan particles according to the following reaction (Eq. (1)):

(1) $$\left[\mathrm{Chitosan}-2{\mathrm{Mg}}^{o}]+{\mathrm{Cu}}^{2+}\to {\mathrm{Mg}}^{2+}+[\mathrm{Chitosan}-{\mathrm{Mg}}^{o}/{\mathrm{Cu}}^{o}\right]$$

The samples were shaken for 5 min and then allowed to stand for 5 min at 25 °C to enable the reduction of Cu^2+^ to Cu^0^. The mixture was passed through 0.2-μm cellulose acetate filter paper by vacuum filtration. In order to get rid of the excess chemicals, the particles were washed several times with deoxygenated double distilled water and rinsed with ethanol and acetone before being dried at 50 °C under vacuum overnight. Finally, the metallized chitosan particles were stored in a closed container for future use. The main properties of the BCP are listed in Table 2.

### 2.4 Experimental design

In order to test the applicability of bi-metalized chitosan particles for the treatment of wastewater containing azo dyes, an adsorption test was done using a river water sample spiked with the orange II dye to 100 mg/L as simulated wastewater. Tests were performed as a batch mode in a 100-mL flask while agitating on a shaker-incubator instrument (Pars Azma Co., Iran). Tests consisted of preparing 50 mL of the azo solution with a given initial concentration and the shaking rate of 100 rpm. The initial pH of the solution was regulated by adding 1 mol/L HCl and NaOH solutions. Aliquots were carefully withdrawn from the solution at different time intervals, and the solution absorbance was measured in the UV–visible range at the maximum absorption (*λ*=483 nm) using a PuXi UV–vis spectrophotometer (TU-1900, China). Centrifugation was done on several samples and shown that the aliquots were particle-free. Thus, the centrifugation of the samples for the taken aliquots was not required. The percentage of dye removal and the adsorption capacity at equilibrium, *q*_e_ (mg/g), by bi-metalized chitosan particles were obtained by using the following equations:

(2) $$OII_{\mathrm{removal}}(\%)=\frac{C_{0}-C_{e}}{C_{0}}\times 100$$

(3) $$q_{e}=\frac{V}{M}(C_{0}-C_{e})$$

where *C*_o_ is the initial orange II dye concentration (mg/L); *C*_e_, the orange II dye concentration at equilibrium (mg/L); *M*, the mass of the adsorbent used (mg); and *V*, the volume of the orange II dye solution used (L).

### 2.5 Adsorption nature

**Fig. 1:**
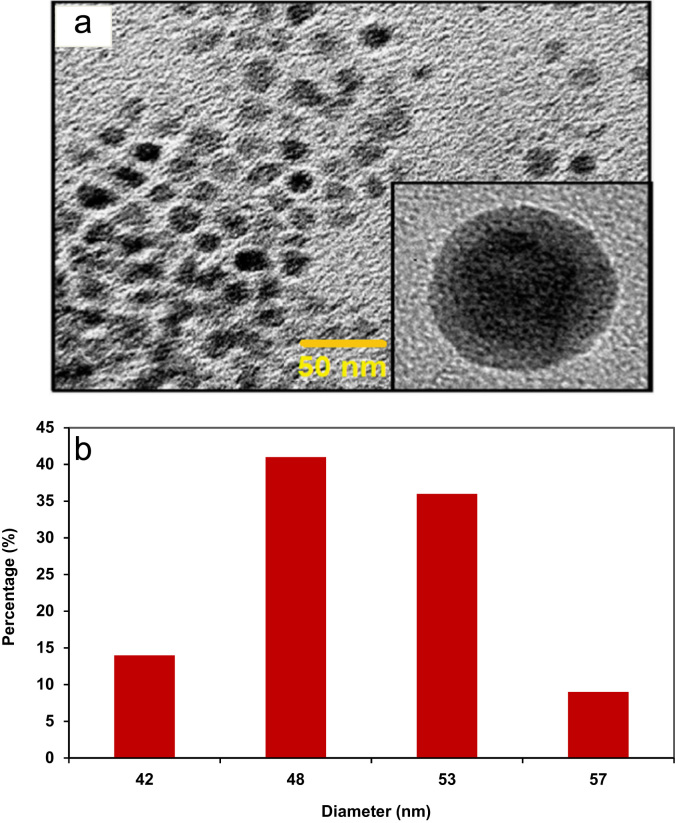
(a) TEM images of freshly bi-metalized chitosan particles, inset is TEM image of an individual Cu/Mg on bi-metalized chitosan particles (b) size distribution of BCP.

The size and the morphology of the bi-metalized chitosan particles were obtained using a transmission electron microscope (TEM) (FEI Tecnai G2 20S TWIN, USA) (Fig. 1). The pH of the zero point charge (pH_zpc_) of the adsorbent was also measured according to the method stated by Ramavandi [5]. The probable degradation intermediates of orange II were identified using a separate HPLC–MS (PerkinElmer Flexar SQ 300 MS, USA) analysis of the treated orange II dye solution.

**Fig. 2:**
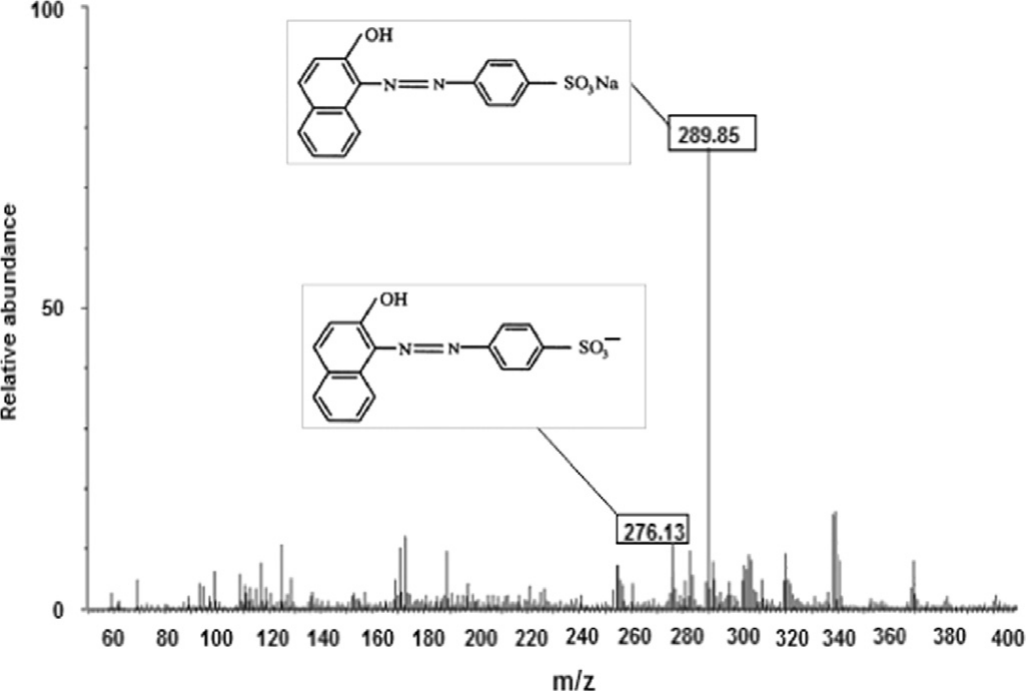
MS spectra of orange II after treatment by bi-metalized chitosan particles.

Orange II dye degradation by bi-metalized chitosan particles, the samples was analyzed by using the HPLC–MS. The HPLC–MS analysis of samples (Fig. 2) shown the presence of two species: residual orange II dye and protonated orange II dye. Therefore, it is implied that the Cu/Mg could not degrade orange II; however, it probably facilitated the adsorption of orange II onto bi-metalized chitosan particles. This also revealed that the main mechanism in orange II removal by bi-metalized chitosan can account for the adsorption process.

## Figures and Tables

**Table 1:** Main properties of orange II dye.

Parameter	Character/value
Molecular structure	
CAS number	633–96–5
Molecular formula	C_16_H_11_N_2_NaO_4_S
Molar mass	350.32 g/mol
Solubility in water	116 g/L at 30 °C
Dissociation constant (pK_a_)	10.65
*λ*_max_ (nm)	485
Color index number	15,510
Dye class	Azo (monoazo)

**Table 2:** The characteristics of bi-metalized chitosan particles.

Parameter	Unit	Value
BET	m^2^/g	12.69
Total pore volume (*P*/*P*_0_=0.990)	cm^3^/g	0.198
Mean pore diameter	nm	49
Pores structure	–	Meso and macro-porous
pH_zpc_	–	6.6
Particle size	nm	42–57
